# Molecular Epidemiological Characteristics of Mycobacterium abscessus Complex Derived from Non-Cystic Fibrosis Patients in Japan and Taiwan

**DOI:** 10.1128/spectrum.00571-22

**Published:** 2022-04-21

**Authors:** Mitsunori Yoshida, Jung-Yien Chien, Kozo Morimoto, Takeshi Kinjo, Akio Aono, Yoshiro Murase, Keiji Fujiwara, Yuta Morishige, Hiroaki Nagano, Ruwen Jou, Naoki Hasegawa, Manabu Ato, Yoshihiko Hoshino, Po-Ren Hsueh, Satoshi Mitarai

**Affiliations:** a Department of Mycobacteriology, Leprosy Research Centre, National Institute of Infectious Diseases, Tokyo, Japan; b Pulmonary and Critical Care Medicine, Department of Internal Medicine, National Taiwan University Hospital, Taipei, Taiwan; c Respiratory Disease Center, Fukujuji Hospital, Japan Anti-Tuberculosis Association, Tokyo, Japan; d Department of Infectious, Respiratory, and Digestive Medicine, Graduate School of Medicine, University of the Ryukyus, Okinawa, Japan; e Department of Mycobacterium Reference and Research, the Research Institute of Tuberculosis, Japan Anti-Tuberculosis Association, Tokyo, Japan; f Department of Respiratory Medicine, Okinawa Chubu Hospital, Okinawa, Japan; g Reference Laboratory of Mycobacteriology, Centers for Disease Control, Ministry of Health and Welfare, Taipei, Taiwan; h Center for Infectious Diseases and Infection Control, School of Medicine, Keio University, Tokyo, Japan; i Departments of Laboratory Medicine and Internal Medicine, National Taiwan University Hospital, National Taiwan University College of Medicine, Taipei, Taiwan; j Departments of Laboratory Medicine and Internal Medicine, China Medical University Hospital, School of Medicine, China Medical University, Taichung, Taiwan; University Paris-Saclay, AP-HP Hospital Antoine Béclère, Service de Microbiologie, Institute for Integrative Biology of the Cell (I2BC), CEA, CNRS

**Keywords:** non-tuberculous mycobacteria, *Mycobacterium abscessus*, non-cystic fibrosis, molecular epidemiology

## Abstract

Mycobacterium abscessus complex (MABC) is a group of emerging, highly antimicrobial-resistant non-tuberculous mycobacteria. Specific MABC clones are spreading globally in patients with cystic fibrosis (CF); however, associated genomic epidemiology is lacking in East Asia, with very few patients with CF. Here, we investigated MABC populations derived from non-CF patients in Japan and Taiwan. Analysis of whole-genome sequencing data of 220 MABC isolates revealed that 112, 105, and 3 were M. abscessus subsp. *abscessus* (ABS), M. abscessus subsp. *massiliense* (MAS), and M. abscessus subsp. *bolletii* (BOL), respectively. Moreover, >50% of ABS and >70% of MAS were related to four predominant clones in the region. Known mutations conferring macrolide resistance were rare (1.4%) and were not enriched in the predominant clones. Conversely, the macrolide-susceptible *erm*(41) T28C mutation was significantly enriched in one predominant ABS clone. The most predominant ABS clone was genetically related to the previously described dominant circulating clone (DCC)1 in patients with CF, whereas no isolates were related to DCC2; isolates related to DCC3 were not necessarily predominant in our sample set. We found that the *erm*(41) T28C mutants spread globally, and some of them reacquired the functional *erm*(41) gene through both point mutation and recombination. This study revealed predominant MABC clones in Japan and Taiwan and their relationship with the globally superadding clones in the patient community with CF. Our study provides insights into the genetic characteristics of globally dominant and area-specific strains isolated from patients with or without CF and differences between globally spread and regionally specific strains.

**IMPORTANCE** Members of Mycobacterium abscessus complex (MABC) are frequently isolated from patients. Studies have reported that predominant clones of MABC (known as dominant circulating clones; DCCs) are distributed worldwide and transmitted from humans to humans in patients with cystic fibrosis (CF). However, associated genomic epidemiology has not yet been conducted in East Asia, including Japan and Taiwan, where there are only a few patients with CF. Using whole-genome sequencing data derived from non-CF patients in Japan and Taiwan, we revealed prevalent clones and the incidence of macrolide resistance-associated mutations in the MABC population in this region. We also clarified the associations between these predominant clones and DCCs in the global CF patient community. Our results would assist further studies in elucidating the genetic characteristics of strains isolated from patients with or without CF, the differences between globally spread and regionally specific strains, and the adaptive evolution of MABC within the host.

## INTRODUCTION

The Mycobacterium abscessus complex (MABC) comprises frequently isolated non-tuberculous mycobacteria (NTM) in patients with or without host risk factors such as cystic fibrosis (CF), bronchiectasis, and other immunocompromised statuses (1–3). The occurrence of pulmonary MABC infection has been investigated, particularly among patients with CF, primarily throughout Europe and America (1, 4, 5), and has also been reported among non-CF patients in some Asian countries (6–10).

The MABC is a triad of rapidly growing NTM comprising M. abscessus subsp. *abscessus* (ABS), M. abscessus subsp. *bolletii* (BOL), and M. abscessus subsp. *massiliense* (MAS) (11). The clinical differences among the subspecies in terms of incidence, manifestation, and prognosis are gradually being elucidated, and they reflect the clinical importance of subspecies differences (12–14). Epidemiological studies on MABC isolates have been conducted at different scales, investigating outbreaks within a single hospital (15, 16), nationwide (17, 18), or with intercontinental spread (19–21). Using whole-genome sequencing (WGS) data of geographically diverse MABC isolated from patients with CF, Bryant et al. revealed that most isolates form dense clusters with low genetic diversity, and three dominant circulating clones (DCCs) were identified, namely, 2 ABS clones (DCC1 and DCC2) and 1 MAS clone (DCC3) (19). Subsequent epidemiological studies using WGS at CF centers in other cohorts confirmed the presence of ABS and MAS clones, widely distributed among the patients studied (17, 18, 22). However, the application of these findings to MABC isolated from non-CF patients and DCC prevalence in East Asian countries, including Japan and Taiwan, where only a few patients are diagnosed with CF, remains unclear (23, 24).

Each subspecies exhibits different susceptibility to macrolides, which are key antibiotics in MABC infection treatment. Of the three subspecies of MABC, nearly all MASs are susceptible to macrolides owing to the presence of the truncated unfunctional erythromycin ribosomal methylase (*erm*)(41) gene (25, 26). The remaining two subspecies contain the functional *erm*(41) gene, which induces macrolide resistance via methylation of the target; however, they become macrolide-susceptible when *erm*(41) loses function through T to C substitution at position 28 (27, 28). In addition to intrinsic macrolide resistance, MABC can acquire mutational macrolide resistance through substitutions in the *rrl* gene encoding the 23S rRNA (29, 30). As these mutations likely affect long-term treatment and promote poor outcomes of MABC infection compared to other NTM diseases (31), detailed epidemiological analyses of macrolide resistance-associated mutations are required. However, the incidence of these mutations in MABC populations derived from non-CF untreated patients in East Asian countries and the relationship between these mutations and the predominant clones are poorly understood.

In this study, we sequenced the genome of 220 MABC clinical isolates obtained from non-CF patients before treatment in four hospitals in three East Asian locations (Tokyo, Okinawa, and Taiwan) covering subtropical to temperate climate zones over 7 years. Using the data set, we examined subspecies distribution, macrolide resistance-associated mutation incidence, and prevalent clones and their genetic features to determine the epidemiological distribution of MABC in Japan and Taiwan.

## RESULTS

### Distribution of MABC subspecies in non-CF patients in Japan and Taiwan.

**FIG 1:**
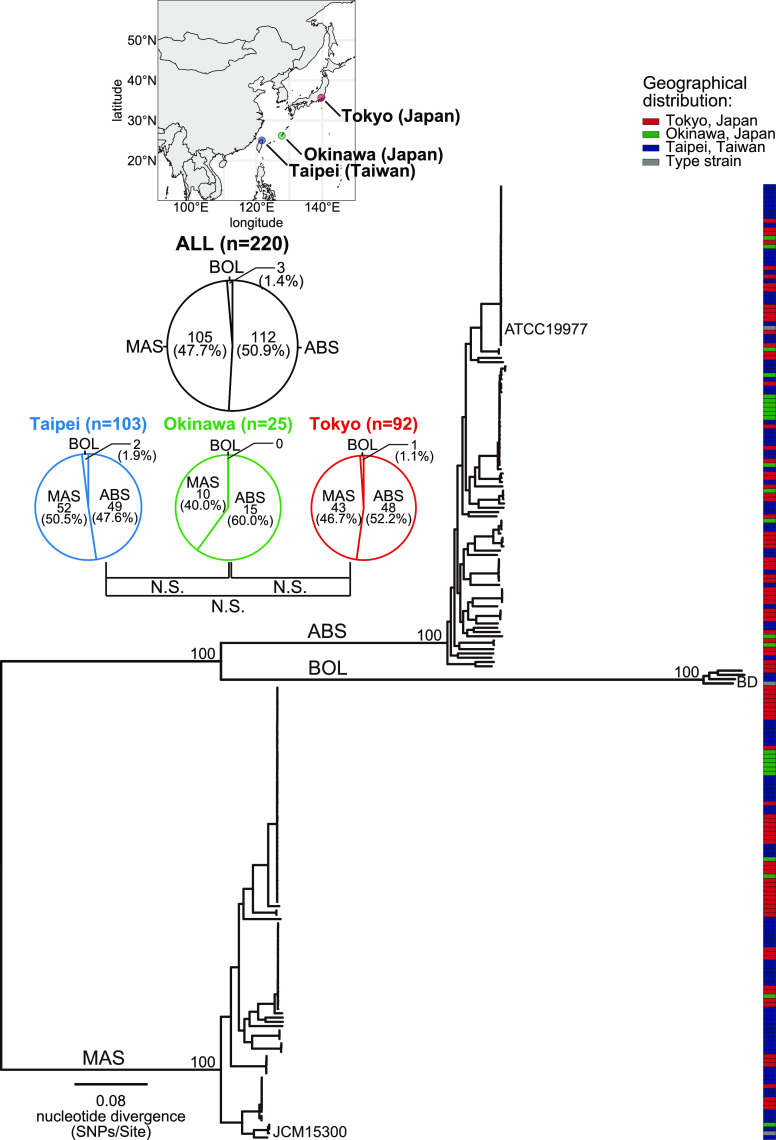
Phylogeny of 220 clinical isolates of MABC in Japan and Taiwan. Core-genome alignment of 220 isolates and 3 reference strains (ABS ATCC19977, MAS JCM15300, and BOL BD) of MABC was generated. A complete genome sequence of ATCC19977 was used as a reference. An alignment containing 235,540 recombination-free variable positions was used with RAxML to construct a maximum likelihood tree with 300 bootstrap replicates. Bootstrap values for the major nodes are shown. Scale bar indicates the mean number of nucleotide substitutions per site (SNPs/Site) on the respective branch. The red, green, and blue boxes indicate the location where each clinical isolate was obtained, and a gray box indicates the reference strain (ATCC19977). Pie charts indicate the ratio of the three subspecies of isolates identified in all (*n* = 220), Tokyo (Japan, *n* = 92), Okinawa (Japan, *n* = 25), and Taipei (Taiwan, *n* = 103), respectively.

We first identified subspecies of 220 MABC clinical isolates based on WGS data to examine the subspecies distribution in Japan and Taiwan. Of the 220 isolates, 112 (50.9%), 105 (47.7%), and 3 (1.4%) were identified as ABS, MAS, and BOL, respectively (Fig. 1). This was confirmed by calculating the species and subspecies boundaries of the MABC clinical isolates using the average nucleotide identity (ANI) values among the MABC isolates (Fig. S1). The minimum ANI among MABC isolates was 96.4%, all ANI values within the three subspecies were above 98% (minimum: 98.1%), and all ANI values between subspecies were below 98% (maximum: 97.6%) (Fig. S1), which are consistent with previous results (14). We detected no significant difference in the composition of MABC subspecies among Tokyo, Okinawa, and Taiwan (Fig. 1).

### Prevalent clones of ABS and MAS in non-CF patients in Japan and Taiwan (East Asia, EA).

**FIG 2:**
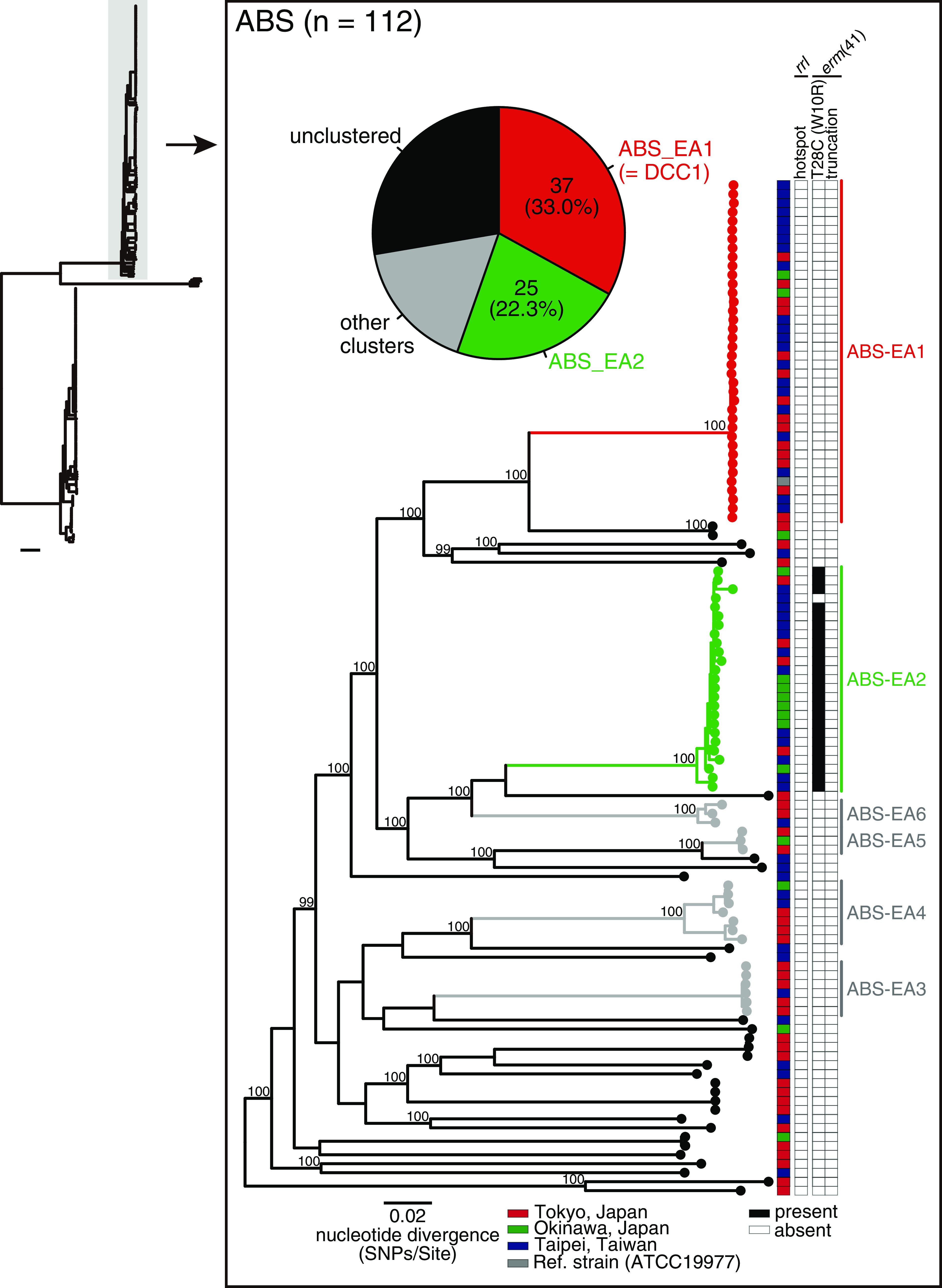
Clustering analysis of ABS in Japan and Taiwan and mutations associated with inducible or acquired macrolide resistance. A core-genome alignment of 112 ABS clinical isolates and a reference strain ATCC19977 was generated (=3,963,788 bp, covering 78.2% of the reference genome). The alignment containing 76,114 recombination-free variable positions within the core genome was used to construct a maximum likelihood tree with 300 bootstrap replicates. Bootstrap values > 98% for the major nodes are shown. Six monophyletic clusters (ABS-EA1 to ABS-EA6) identified using TreeGubbins are shown. The pie chart indicates the proportion of the identified clusters, and the two dominant clusters (ABS-EA1 and ABS-EA2) are depicted in red and green, respectively. The location where each clinical isolate was isolated is indicated, as shown in Fig. 1. The presence (black) and absence (white) of macrolide resistance-associated mutations are indicated. Scale bar; the mean number of nucleotide substitutions per site (SNPs/Site) on the respective branch.

**FIG 3:**
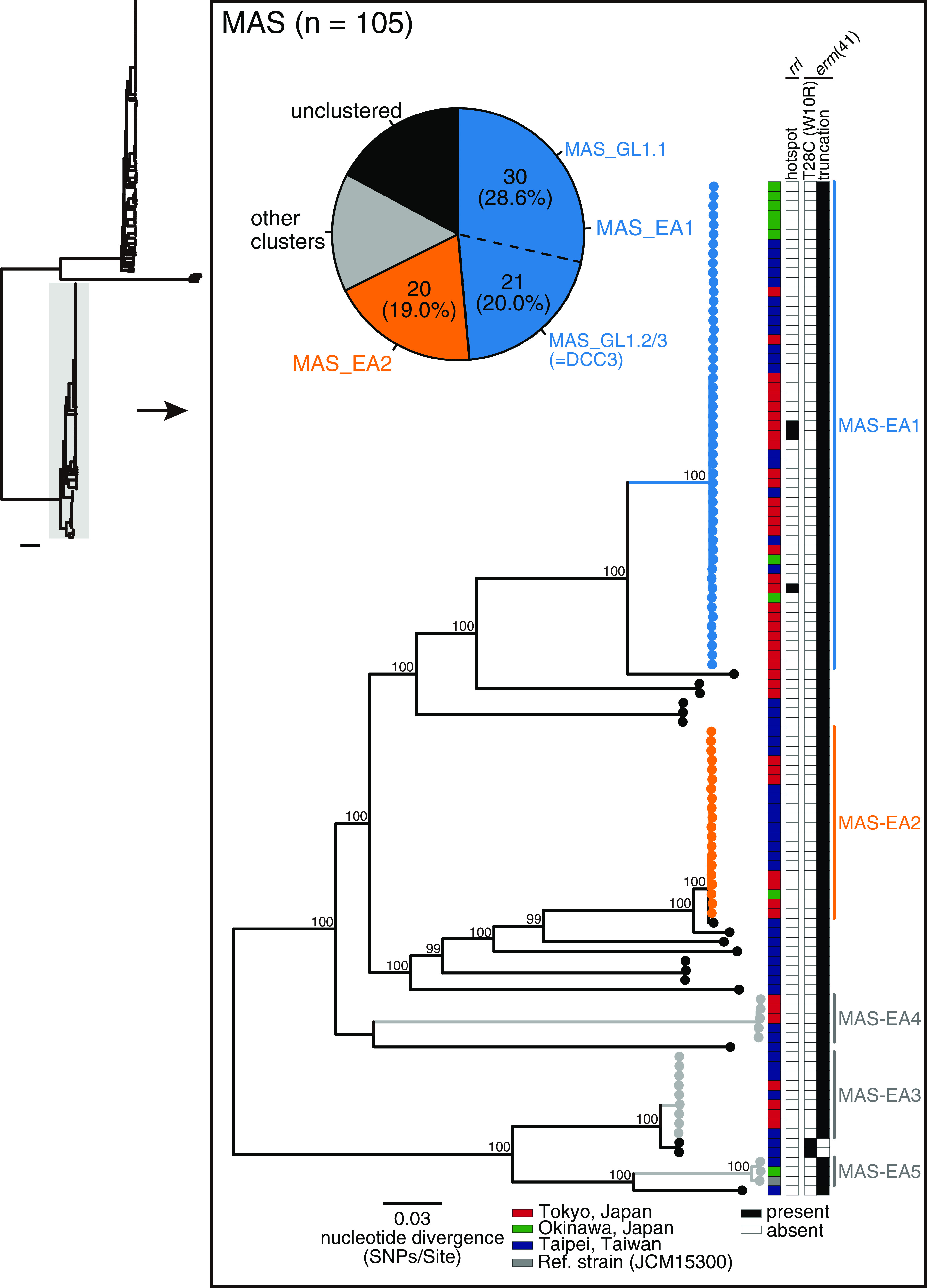
Clustering analysis of MAS in Japan and Taiwan and mutations associated with inducible or acquired macrolide resistance. A core-genome alignment of 105 MAS clinical isolates and a reference strain JCM15300 was generated (=4,033,769 bp, covering 81.0% of the reference genome). The alignment containing 48,718 recombination-free variable positions located within the core-genome was used to construct a maximum likelihood tree with 300 bootstrap replicates. Bootstrap values > 98% for the major nodes are shown. Five monophyletic clusters (MAS-EA1 to MAS-EA5) identified using TreeGubbins are shown. The pie chart indicates the proportion of identified clusters, and the two dominant clusters (MAS-EA1 and MAS-EA2) are depicted in blue and orange, respectively. The location where each clinical isolate was isolated and the presence (black) and absence (white) of macrolide resistanceassociated mutations are the same as those in Fig. 2. Scale bar; the mean number of nucleotide substitutions per site (SNPs/Site) on the respective branch.

To further investigate the genetic relatedness of isolates from individual patients in Japan and Taiwan, we analyzed the phylogeny of ABS and MAS separately (Fig. 2, Fig. 3). Among the 112 ABS isolates, we identified six clusters (ABS-EA1 to ABS-EA6, shown in Fig. 2), including 85 isolates (75.9%). Of these isolates, 37 (33.0%) and 25 (22.3%) from the three locations belonged to the most prominent clusters (ABS-EA1 and ABS-EA2, respectively) (Fig. 2). The other three ABS-EA clusters (ABS-EA3, ABS-EA5, and ABS-EA6) consisted of isolates from two different sites (Tokyo/Okinawa, Tokyo/Taipei, or Taipei/Okinawa). We identified five clusters of 105 MAS isolates, including 87 isolates (82.9%) (MAS-EA1 to MAS-EA5, shown in Fig. 3). Of these clustered isolates, 51 (48.6%) and 20 (19.0%) from the three locations belonged to the first (MAS-EA1) and second (MAS-EA2) most predominant clusters (Fig. 3), respectively, whereas the other three clusters (MAS-EA3, MAS-EA4, and MAS-EA5) consisted of isolates from two locations. We also examined the population differences between ABS and MAS in this region. The genome-wide nucleotide diversity of MAS was significantly higher than that of ABS (Fig. S2A, *P < *5.1e-06), and the total number of genes within MAS isolates was larger than that of the ABS isolates (Fig. S2B); pairwise genetic distances between isolates in each MAS-EA cluster (ranged from 8 to 142 SNPs) were significantly lower those in each cluster of ABS-EA cluster (ranged from 4 to 1444 SNPs) (Fig. S2C).

### Mutations associated with macrolide resistance of ABS/MAS in non-CF patients in Japan and Taiwan.

We determined the incidence of genetic mutations associated with macrolide resistance. None of the ABS isolates harbored mutations in the *rrl* hot spot (2269–2271 bp in the *rrl* gene of ATCC19977) (Fig. 2). Among the 112 ABS, 24 (21.4%) carried the macrolide-susceptible *erm*(41) T28C sequence variant. Notably, all *erm*(41) T28C sequences belonged to ABS-EA2 and exhibited significant enrichment in this clade (Fig. 2, *P < *2.2e-16). Three of the 105 MAS (2.9%) had mutations in the hot spot on *rrl*, all of which belonged to MAS-EA1; however, they were not significantly enriched (Fig. 3, *P = *0.24). Nearly all MAS (103 isolates) had truncation of *erm*(41) without substitution in the hot spot of *rrl*. However, two MAS (TWN-024 and TWN-041) exceptionally displayed a full-length *erm*(41), with a simultaneous T28C mutation. A total of 102 MAS isolates (97.1%) were predicted to be susceptible to macrolides. Together with the fact that no BOL (*n* = 3) displayed substitutions in the *rrl* hot spot or T28C mutation in *erm*(41) (data not shown), 126/220 MABC isolates (57.3%, 24 ABS and 102 MAS) were predicted to be susceptible to macrolides due to dysfunctional *erm*(41), whereas 91 isolates (41.4%, 88 ABS and 3 BOL) may induce macrolide resistance, and the remaining three isolates (1.4%) carried mutations that have been reported to confer macrolide resistance.

### Association between ABS prevalent in non-CF patients in Japan and Taiwan and previously described DCCs in global CF patients.

**FIG 4:**
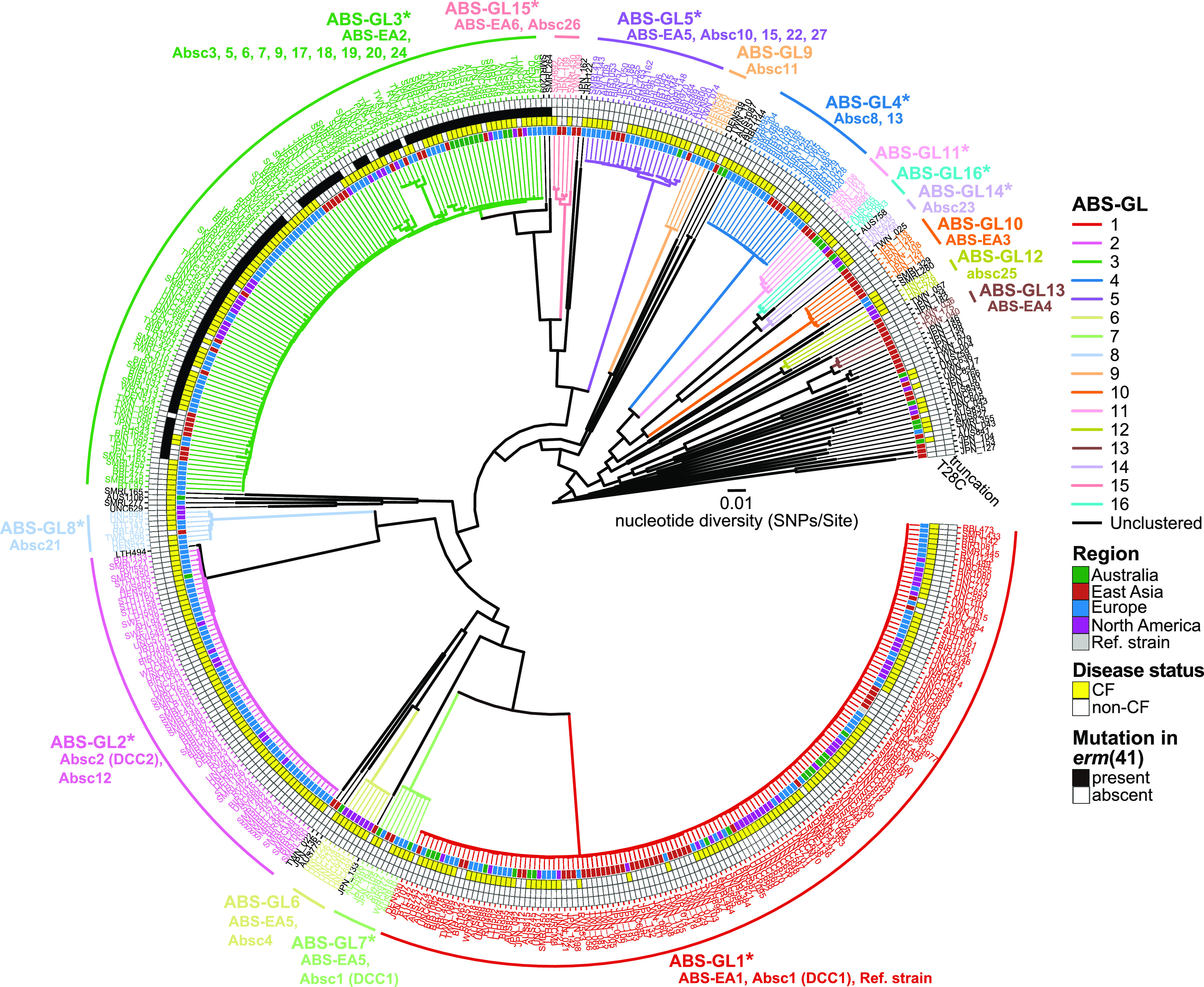
Phylogenetical association between ABS isolates in Japan and Taiwan and those in other countries. Phylogeny of ABS from several countries was estimated using a core-genome alignment of 461 clinical isolates from four regions (Australia, East Asia, Europe, and North America). The complete genome sequence of ATCC19977 was used as a reference. The lignment containing 102,613 recombination-free variable positions in the core genome (3,584,621 bp, covering 70.7% of the reference genome) was used with RAxML to construct a maximum likelihood tree with 300 bootstrap replicates. The 16 monophyletic clusters (ABSGL1 to ABS-GL16) identified using TreeGubbins, and the corresponding clusters identified in Fig. 2, and Bryant et al. (2016) are shown. The presence (black) and absence (white) of inducible macrolide resistance-associated mutations in the *erm*(41) gene are indicated. Disease status (CF; yellow and non-CF; white) of the corresponding patients are shown. Each color box corresponds to the region where the clinical isolate was isolated. Asterisks indicate clusters that consist of isolates from more than two regions. Scale bar indicates the mean number of nucleotide substitutions per site (SNPs/Site) on the respective branch.

To analyze the associations between prevalent ABS clones in East Asian countries and previously described DCCs, publicly available data of 349 ABS from individual CF patients (Table S1) (19) were combined with our data set. In the global ABS population, we identified 16 clusters (ABS-GL1 to ABS-GL16 shown in Fig. 4), of which 10 (ABS-GL1, 3, 4, 5, 7, 8, 11, 14, 15, and 16) consisted of isolates from both CF and non-CF patients in more than two regions (Fig. 4). Two clusters (ABS-GL10 and ABS-GL13) exclusively consisted of isolates from non-CF patients in East Asia, and three clusters (ABS-GL6, ABS-GL9, and ABS-GL12) consisted of isolates from CF patients in the US or Europe. In ABS-GL1, all ABS-EA1 isolates, which is the most predominant ABS clone in Japan and Taiwan (Fig. 2), were clustered with isolates that were identified as DCC1 (=Absc1) within the global CF patient community (19) (Fig. 4). Although all ABS identified as DCC2 (=Absc2) (19) were exclusively clustered in ABS-GL2, no isolate from non-CF patients in Japan and Taiwan belonged to this cluster (Fig. 4). In ABS-GL3, all ABS-EA2 isolates were clustered with multiple Absc clusters derived from CF patients in several regions, and 97 out of 114 isolates (85.1%) harbored *erm*(41) T28C mutation (Fig. 4).

### Reacquisition of functional *erm*(41) gene in ABS-GL3 isolates.

**FIG 5:**
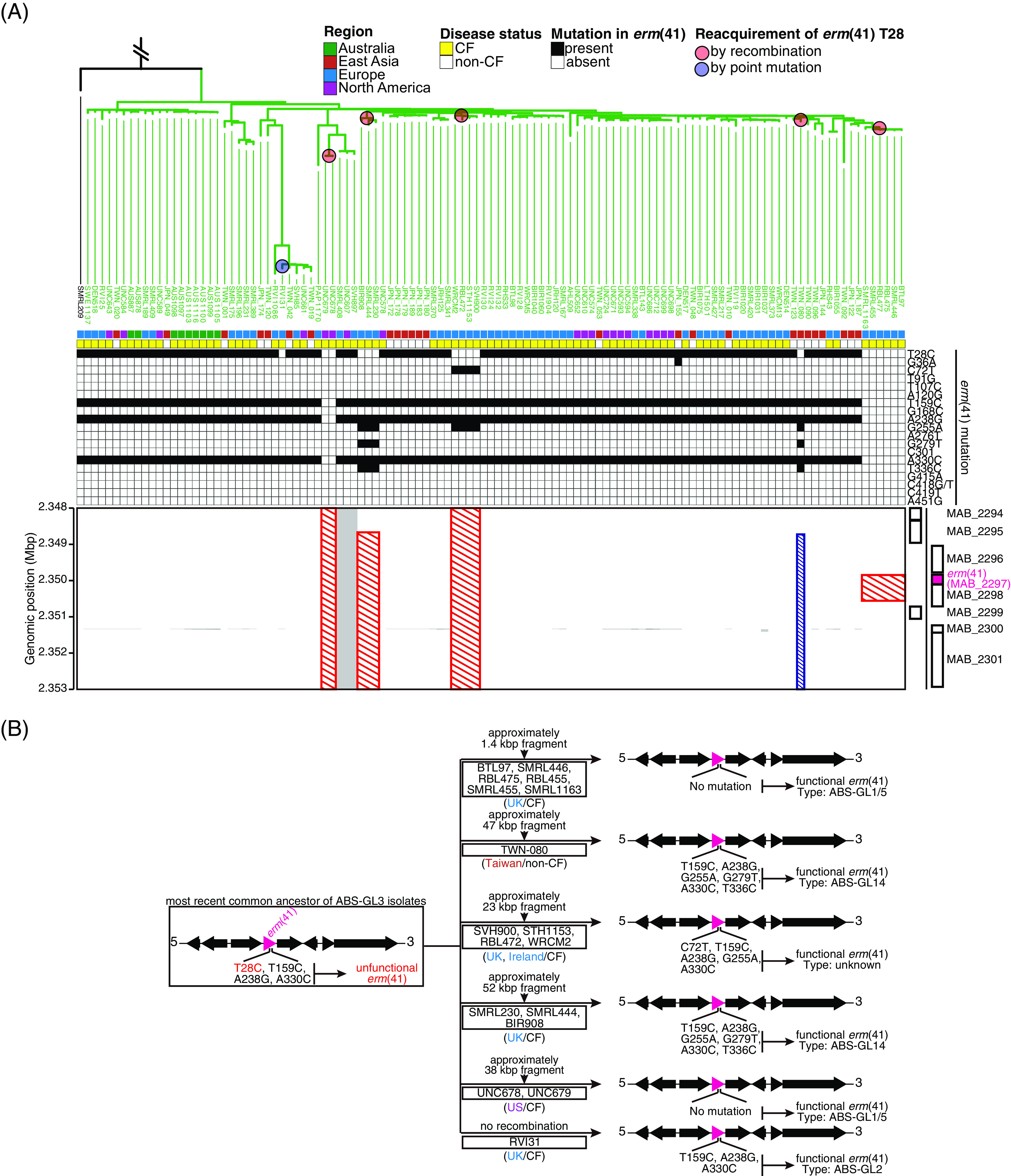
Reacquisition of wild-type T28 genotype in *erm*(41) of ABS clinical isolates belonged to ABS-GL3. (A) Recombination events in the genomic region around the *erm*(41) gene of ABS clinical isolates belonged to the ABS-GL3 cluster. Red or blue shaded boxes indicate common or sporadic recombination events. The presence and absence of mutations in the *erm*(41) gene, disease status (CF or non-CF) of corresponding patients, and the region where the clinical isolate was isolated are shown as Fig. 4. (B) A schematic depiction regarding the reversion of the wild-type *erm*(41) T28 genotype. Black and magenta arrows indicate genes, the fourth of which is *erm*(41).

Although ABS-GL3 isolates exclusively displayed the *erm*(41) T28C mutation, it appeared that isolates of several sub-clades within this cluster reversed to the wild-type T28 genotype in *erm*(41) (Fig. 5A, Fig. S3). To explore the mode of reacquisition of the T28 genotype, we examined the mutation pattern of *erm*(41) in all ABS clinical isolates (Fig. S3). We found that the ancestral form of *erm*(41) in clinical isolates belonging to ABS-GL3 carries the T159C, A238G, and A330C sequence variants in addition to T28C (Fig. 5A, Fig. S3). However, isolates belonging to the four sub-clades and one isolate (TWN-080) that reversed the T28 genotype showed distinctly different mutation patterns (red circles in Fig. 5A). In these isolates, fragments harboring the wild-type *erm*(41) T28 genotype were inserted via recombination around *erm*(41) (red and blue shaded boxes in Fig. 5A, Fig. 5B). It was also observed that ABS-GL3 isolates showed phylogenetically high recombination potential than other clinical isolates (Fig. S3). In contrast, *erm*(41) from an isolate (RVI31, blue circles in Fig. 5A) displayed the same mutation pattern as the ancestral type, with the exception of wild-type T28, and no recombination event was detected in this genomic region of RVI31 (Fig. 5A and B).

### Association between MAS prevalent in non-CF patients in Japan and Taiwan and previously described DCCs in global CF patients.

**FIG 6:**
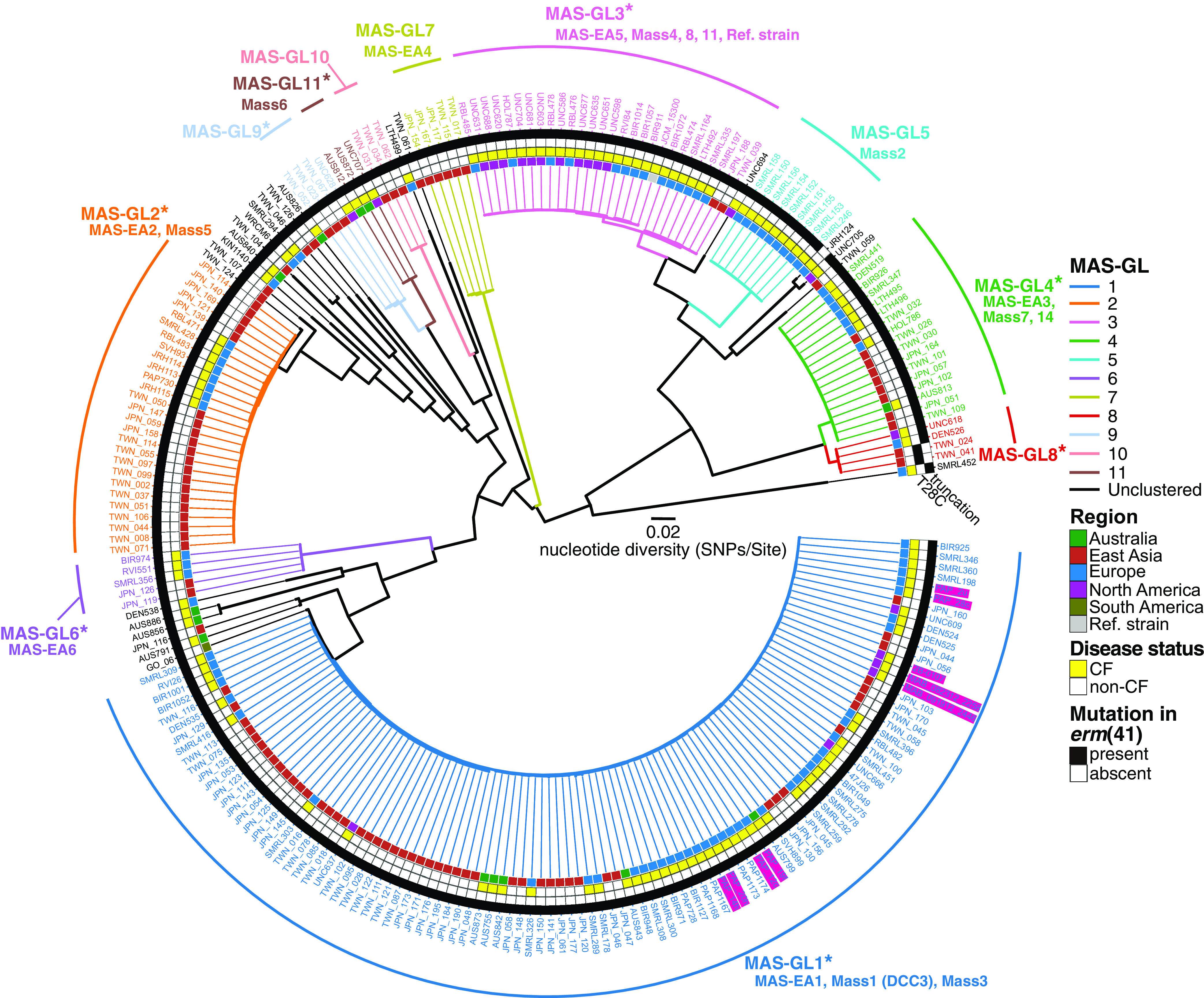
Phylogenetical association between MAS in Japan and Taiwan and those in other countries. Phylogeny of global MAS was estimated using core-genome alignment of 233 clinical isolates from five regions (Australia, East Asia, Europe, North America, and South America). The complete genome sequence of JCM15300 was used as a reference. The alignment containing 58,975 recombination-free variable positions located in the core genome (3,789,583, covering 76.1% of the reference genome) was used with RAxML to construct a maximum likelihood tree with 300 bootstrap replicates. The 11 monophyletic clusters (MASGL1 to MAS-GL11) identified using TreeGubbins, and the corresponding clusters identified in Fig. 3, and Bryant et al. (2016) are shown. In Fig. 4, the presence and absence of mutations in the *erm*(41) gene, disease status (CF or non-CF) of the corresponding patients, and the region where the clinical isolate was isolated are shown. Asterisks indicate clusters that consist of isolates from more than two regions. Magenta boxes indicate clinical isolates that caused nosocomial outbreaks of MAS (15, 32). Scale bar indicates the mean number of nucleotide substitutions per site (SNPs/Site) on the respective branch.

**FIG 7:**
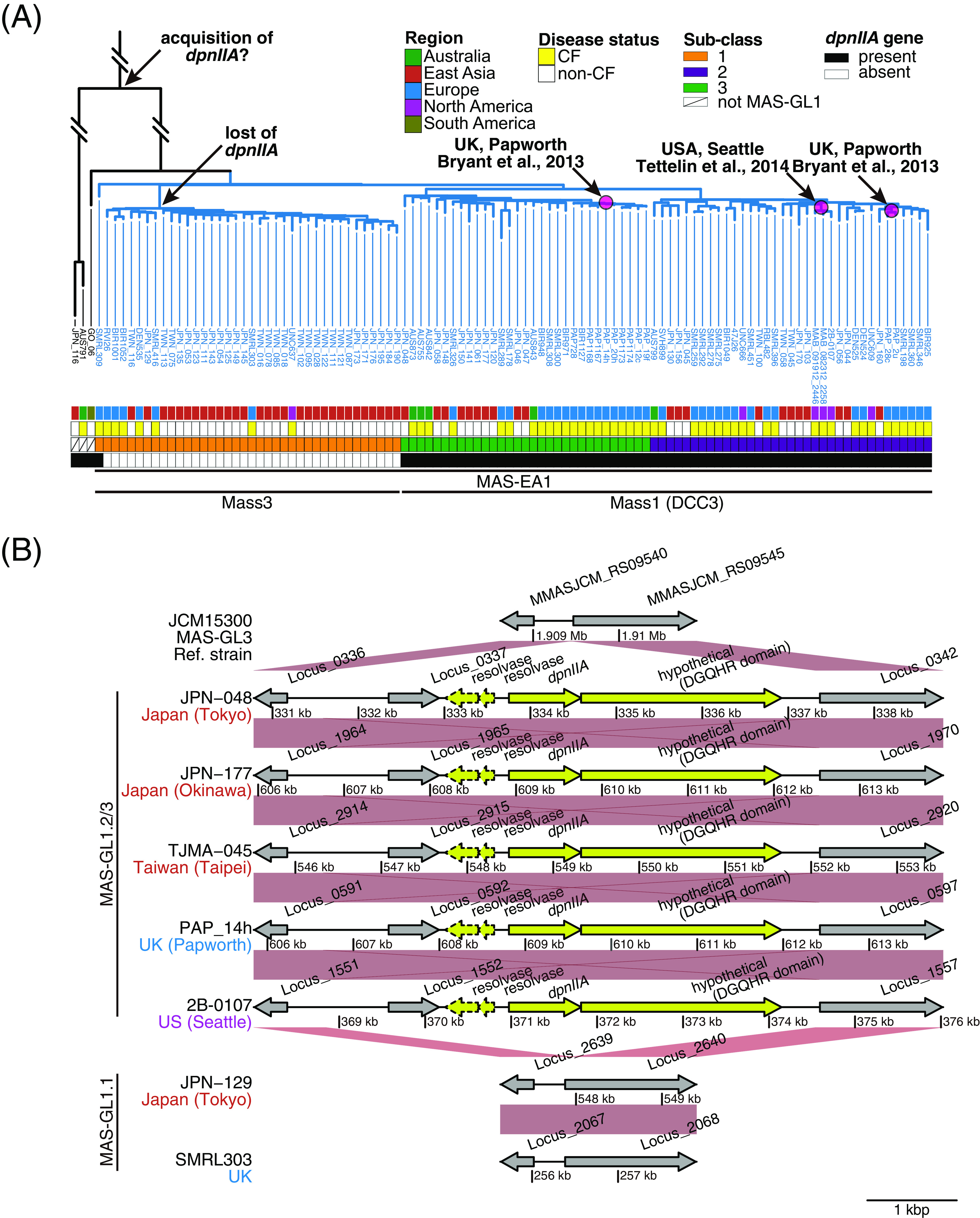
Genetic components associated with MAS isolates that were genetically related to previously reported outbreak strains. (A) Sub-cluster analysis of MAS belonged to the MASGL1 cluster. The phylogenetic tree of MAS clinical isolates was constructed as described in Fig. 6. Sub-clusters were inferred using fastBAPS (47) with default settings, in which the coregenome alignment of clinical isolates belonging to MAS-GL1 was used as an input file. The presence (black) and absence (white) of the *dpnIIA* gene, disease status (CF or non-CF) of the corresponding patients, and the region where the clinical isolate was isolated are shown. (B) Examples of acquisition of the *dpnIIA* locus between clinical isolates belonged to MAS-GL1.2 and MAS-GL1.3. Arrows indicate genes annotated with DFAST-core (48), and dotted arrows indicate pseudogenes estimated by DFAST-core. Orthologous genes between clinical isolates are shown with red connections and are plotted with genoPlotR (49). Five genes exclusively associated with clinical isolates that belonged to MAS-GL1.2 and MAS-GL1.3 are colored in yellow.

In addition to ABS isolates, we assembled publicly available WGS data for 127 MAS clinical isolates from patients with CF from seven countries (listed in Table S1) (15, 19, 32) and combined them with our data set. We identified 11 clusters (MAS-GL1 to MAS-GL11 shown in Fig. 6), of which eight (MAS-GL1, 2, 3, 4, 6, 8, 9, and 11) could be considered as globally dispersed clones in both CF and non-CF patients. In contrast, MAS-GL7 and MAS-GL10 consisted of isolates from non-CF patients in East Asia, and MAS-5 consisted of isolates from CF patients in western countries, respectively. MAS-GL1 contained all MAS-EA1 isolates, which is the most predominant MAS clone in Japan and Taiwan (Fig. 3), and previously described DCC3 (=Mass1) and Mass3 from CF patients (19) (Fig. 6, Fig. 7A). MAS-GL2 contained all MAS-EA2 isolates, and it is identified as a globally spreading clone Mass5 (19). Although TWN-024 and TWN-041 carrying full-length *erm*(41) were clustered with DEN526 (from Denmark) and UNC618 (from the USA) in MAS-GL8, the latter two isolates showed truncation of *erm*(41), similar to all other MAS isolates (Fig. 6). One isolate (JRH124 from the UK) did not belong to any MAS-GL cluster and sporadically displayed full-length *erm*(41) without T28C substitution (Fig. 6).

### MAS isolates from non-CF patients in Japan and Taiwan that are genetically related to MAS from patients during previous outbreaks at CF centers.

Notably, MAS-GL1 contained isolates from patients during outbreaks in two CF centers (15, 32) (magenta boxes in Fig. 6). In our sample set, half of the MAS isolates belonged to MAS-GL1 (MAS-EA1). These observations prompted us to investigate MAS-GL1 phylogeny further. We identified three sub-clusters within MAS-GL1 (MAS-GL1.1 to MAS-GL1.3, Fig. 7A) and found that DCC3 (Mass1) isolates and isolates from patients during previous outbreaks belonged exclusively to MAS_GL1.2 or MAS-GL1.3 (Fig. 7A). In MAS clinical isolates from non-CF patients in Japan and Taiwan, 21 out of 105 (20%) belonged to MAS_GL1.2 or MAS-GL1.3, whereas 30 (28.6%) belonged to MAS-GL1.1 (Fig. 3). To genetically characterize the MAS isolates that belonged to MAS_GL1.2 or MAS-GL1.3, we identified four genes that were significantly associated with these clades (*P < *2.7e-52 after Bonferroni correction, Fisher's exact test, Table S2). These genes included two resolvases, both of which may be unfunctional due to frameshift, a DGQHR domain-containing hypothetical protein, and a site-specific DNA-adenine methyltransferase *DpnIIA* (Fig. 7B). The most recent common ancestor of MAS-GL1 retained the *DpnIIA* gene, and a descendant lineage leading to DCC3 also retained this gene. Meanwhile, another descendant lineage leading to Mass3 was determined to have lost this gene.

## DISCUSSION

Herein, we investigated the epidemiological distribution of pulmonary MABC infection in Japan and Taiwan based on WGS data from a set of clinical isolates obtained from non-CF pulmonary MABC patients. This is the first WGS-based epidemiological study focusing on MABC clinical isolates from non-CF patients in East Asia, expanding the previous findings reported for patients with CF in Western countries (15–17, 19, 33). Half of our sample set consisted of ABS, whereas the other half was MAS and a small number of BOL. No geographical differences were noted in the subspecies composition of the three locations examined, consistent with the findings of previous epidemiological studies on non-CF patients conducted using conventional methods in Japan and other East Asian cohorts (10, 34, 35). While the incidence of BOL was very low in our cohort, BOL reportedly accounts for 10%–20% of MABC in European cohorts of patients with CF (22, 36). Although the composition of the three subspecies is difficult to compare directly as it primarily depends on the clinical and environmental settings of each institution, these observations may signify differences in the geographic distribution of BOL and/or the susceptibility to BOL between CF and non-CF patients.

As macrolides are crucial in pulmonary MABC infection treatment, we revealed the incidence of macrolide resistance-associated mutations in our sample set. Although all our clinical isolates were derived from untreated patients, 1.4% of the isolates were expected to acquire macrolide resistance through mutations in *rrl*, and these mutations did not enrich any dominant clones of MABC in Japan and Taiwan. Our analysis suggested that at least 57% of MABC clinical isolates were susceptible to macrolides owing to the loss of function of *erm*(41), suggesting that macrolide-containing regimens are effective in treating more than half of MABC infections in our cohort. Notably, over 20% of ABS carried the macrolide-susceptible *erm*(41) T28C mutation, and this mutation was significantly enriched in one of four dominant clones of MABC in this area. Although it remains unknown whether the ABS *erm*(41) T28C sequevar is present in similar proportions elsewhere, these results emphasize the clinical importance of accurate genotyping of *rrl* and *erm*(41) before MABC treatment initiation, as recommended by recent treatment guidelines for pulmonary MABC infection (13).

There were several clusters of isolates from non-CF patients in both Japan and Taiwan, but the number of isolates in each cluster differed. In ABS isolates, 33% and 22% of isolates belonged to the most prominent and second-largest clusters (ABS-EA1 and ABS-EA2), respectively, representing dominant clones in Japan and Taiwan. Remarkably, the *erm*(41) T28C mutation exclusively accumulated in the ABS-EA2 cluster. These results indicate that the ABS *erm*(41) T28C sequevar can be considered a significant clade in Japan and Taiwan. As for MAS, this trend was more pronounced, with more than 70% of MAS isolates belonging to two large clusters (MAS-EA1 and MAS-EA2). These results suggest that specific clones of ABS and MAS are intensively isolated from non-CF patients in Japan and Taiwan. We observed that MAS has higher nucleotide diversity and a larger gene repertoire but shows lower genetic distances among isolates belonging to the identified clusters. These results suggest that MAS is a more genetically diverse population than ABS, and certain homologous clones may adapt to preferentially infect the respiratory system.

Bryant et al. reported that 2 ABS clones (DCC1 and DCC2) and 1 MAS clone (DCC3) are dominantly spreading worldwide (19). Hence, we analyzed the prevalence of these dominant clones in non-CF patients in East Asian regions. Our results indicated that clinical isolates related to DCC1 clones are also common in Japan and Taiwan. However, no clinical isolates related to DCC2 clones were identified in our sample set, and clinical isolates related to DCC3 clones were not necessarily dominant in our sample set. Consistent with our results, recent genomic epidemiological studies have reported that DCC1 clones are predominant in CF cohorts whereas DCC2 and DCC3 are not (18, 22). Thus, it is suggested that the DCC1 clone is distributed worldwide and preferentially infects and causes pulmonary MABC disease over other clones in both CF and non-CF patients. However, it remains unclear whether DCC2 or DCC3 preferentially causes disease compared to other clones. Further genomic, epigenomic, and phenotypic analyses are needed to understand the differences in pathogenicity, as observed in M. tuberculosis (37–39).

Notably, the ABS *erm*(41) T28C isolates from several countries belonged exclusively to ABS-GL3, indicating that this sequevar can be considered as one of the globally spreading clones. Furthermore, some isolates within ABS-GL3 appeared to reverse the wild-type T28 genotype in *erm*(41), consistent with a recent global phylogenomic analysis of MABC (40). Moreover, we identified two patterns of reversion of the wild-type T28 genotype: the reversion of the wild-type *erm*(41) T28 genotype by point mutation and the incorporation of *erm*(41) from isolates belonging to other clusters into ABS-GL3 isolates. As the *erm*(41) T28C mutation is not reported to be detrimental to the survival of the bacterium in macrolide-free environments, one possible explanation for the “genetic throwback” is that the ABS-GL3 isolates have undergone adaptive evolution to survive in the macrolide environment within the host and that the isolates may be infecting other patients indirectly or directly (41). High recombination potential observed in ABS-GL3 isolates, compared to strains in different clades (Fig. S3), may drive the adaptive evolution. We also observed 2 MAS (TWN-024 and TWN-041) isolates carrying full-length *erm*(41) genes with the T28C mutant and 1 MAS isolate carrying full-length *erm*(41). The mutation pattern of *erm*(41) in TWN-024 and TWN-041 was identical to that in ABS-GL3 isolates (T28C, T159C, A238G, and A330C sequence variants, data not shown). These results suggest that horizontal transfer of *erm*(41) that alters macrolide susceptibility may occur, although not frequently, between ABS and MAS.

In this study region, more than half of the MAS isolates belonged to MAS-GL1. This large cluster was divided into three sub-clades, two of which (MAS-GL1.2 and MAS-GL1.3) were exclusively related to isolates from patients during outbreaks in two CF centers in the USA and UK (15, 32) and were annotated as DCC3 (19). Thus, we searched for genes significantly associated with MAS-GL1.2 and MAS-GL1.3. The genes included a functional methyltransferase *dpnIIA* gene, expected to belong to the “DpnII restriction gene cassette.” DpnII only methylates double-stranded DNA, whereas DpnI can methylate both single- and double-stranded DNA, although both enzymes act at 5′-GATC-3′ in DNA (42). A recent study demonstrated that the *dpnIIA* gene of MABC participates in altering the genome-wide methylation and expression levels of several genes and is required for intracellular survival (41). Although other mycobacterial species express the homolog, no additional available functional information on the gene exists. The proportion of isolates related to DCC3 in our cohort was not necessarily higher than those related to other MAS clades. In addition, strains belonging to MAS-GL1.1 and MAS-GL2 (=MAS-EA2, Mass5), which lost *dpnIIA*, were isolated to the same extent as isolates related to DCC3. These results indicate that, at least in our cohort, *dpnIIA* alone does not serve as a determinant of whether a MABC clone is dominant. Nevertheless, all strains isolated during nosocomial outbreaks in CF centers (15, 32) carry *dpnIIA*. Notably, a strain (GO-06) isolated from a nosocomial infection in the surgical site of several patients in Brazil (43) still carries *dpnIIA*, although it is not related to DCC3 (Fig. 7). Further research is needed to determine how the acquisition of *dpnIIA* led to the nosocomial outbreak of MAS and whether there are other cases of outbreaks caused by clade-specific gene acquisition to understand the pathogenic evolution of MABC.

The present study had some limitations. First, this study was conducted using MABC clinical isolates from only four facilities in three locations; therefore, further validation using other sample sets is needed to examine whether it presents the complete picture of pulmonary MABC infection in Japan and Taiwan. Second, there is a lack of information regarding epidemiological links among patients. The analysis of the genetic distances between clinical isolates from individuals showed the possibility of direct or indirect transmission of MABC (Fig. S2C) (15, 16). Further analysis, including tracking of epidemiological linkages among patients and environmental sampling, is required to understand the mode of transmission of MABC. Finally, as only a few patients with CF in East Asia, our sample set did not include clinical isolates derived from patients with CF in Japan and Taiwan. Therefore, it cannot be determined whether the detected area-specific clusters were caused simply by differences in the geographical distribution of MABC or by differences in patients’ disease status (CF or non-CF).

### Conclusions.

We revealed the prevalent clones and the incidence of macrolide resistance-associated mutations in MABC isolated from non-CF patients in Japan and Taiwan. We also clarified the relationship between these clones and previously described dominant clones in the international CF patient community. Our results provide a cornerstone for WGS-based epidemiological analysis of pulmonary MABC disease in the East Asian region. Furthermore, our study provides insights to elucidate the genetic differences between globally predominant and area-specific clones isolated from patients with and without CF.

## MATERIALS AND METHODS

### Sample collection.

Totally, 220 clinical isolates (one isolate per patient before treatment initiation) were recovered from respiratory specimens from 2012 to 2017 (Table S3). All patients met the diagnostic criteria for ATS/ERS/ESCMID/IDSA (13); 117 clinical isolates were obtained from three hospitals in Japan (Fukujuji Hospital, Keio University Hospital, and Okinawa Chubu Hospital), and 103, from a hospital in Taiwan (National Taiwan University Hospital). All isolates were classified as MABC by DDH Mycobacteria (Kyokuto Pharmaceutical Industrial, Tokyo, Japan) and/or MALDI-TOF MS (Bruker Daltonics, MA, USA).

### Genomic analysis.

The raw WGS data of each isolate were *de novo* assembled in the Shovill pipeline (https://github.com/tseemann/shovill) with default settings. Assembly statistics are listed in Table S4. The MUMmer package (44) was used to identify conserved genomic regions among isolates and the SNP sites within these regions, and Gubbins (45) was used to infer recombination sites. RAxML ver. 8.2.12 with the General Time Reversible (GTR)-GAMMA model (46) was used to reconstruct maximum-likelihood trees.

Further details on the methods used are presented in the online data supplement.

### Ethics approval.

This study was reviewed and approved by the Medical Research Ethics Committee of the Fukujuji Hospital (#18038), Keio University Hospital (#20080131), Okinawa Chubu Hospital (#2018-89) and National Taiwan University Hospital (#201808087RINA) for the use of human subjects.

### Data availability.

All raw data and materials are available from the corresponding authors on request. All raw read data for the newly sequenced isolates in this study are available at the NCBI under BioProject accession number PRJDB10566.

## References

[1] Roux A-L, Catherinot E, Ripoll F, Soismier N, Macheras E, Ravilly S, Bellis G, Vibet M-A, Le Roux E, Lemonnier L, Gutierrez C, Vincent V, Fauroux B, Rottman M, Guillemot D, Gaillard J-L, Jean-Louis Herrmann for the OMA Group. 2009. Multicenter study of prevalence of nontuberculous mycobacteria in patients with cystic fibrosis in France. J Clin Microbiol 47:4124–4128. doi:10.1128/JCM.01257-09. 19846643 PMC2786646

[2] Olivier KN, Weber DJ, Wallace RJ, Faiz AR, Lee J-H, Zhang Y, Brown-Elliot BA, Handler A, Wilson RW, Schechter MS, Edwards LJ, Chakraborti S, Knowles MR. 2003. Nontuberculous mycobacteria: I: Multicenter prevalence study in cystic fibrosis. Am J Respir Crit Care Med 167:828–834. doi:10.1164/rccm.200207-678OC. 12433668

[3] Collins FM. 1989. Mycobacterial disease, immunosuppression, and acquired immunodeficiency syndrome. Clin Microbiol Rev 2:360–377. doi:10.1128/CMR.2.4.360. 2680057 PMC358130

[4] Adjemian J, Olivier KN, Prevots DR. 2014. Nontuberculous mycobacteria among patients with cystic fibrosis in the United States: screening practices and environmental risk. Am J Respir Crit Care Med 190:581–586. doi:10.1164/rccm.201405-0884OC. 25068291 PMC4214089

[5] Gardner AI, McClenaghan E, Saint G, McNamara PS, Brodlie M, Thomas MF. 2019. Epidemiology of nontuberculous mycobacteria infection in children and young people with cystic fibrosis: analysis of UK cystic fibrosis registry. Clin Infect Dis 68:731–737. doi:10.1093/cid/ciy531. 29982302 PMC6376093

[6] Namkoong H, Kurashima A, Morimoto K, Hoshino Y, Hasegawa N, Ato M, Mitarai S. 2016. Epidemiology of pulmonary nontuberculous mycobacterial disease, Japan. Emerg Infect Dis 22:1116–1117. doi:10.3201/eid2206.151086. 27191735 PMC4880076

[7] Koh W-J, Kwon OJ, Jeon K, Kim TS, Lee KS, Park YK, Bai GH. 2006. Clinical significance of nontuberculous mycobacteria isolated from respiratory specimens in Korea. Chest 129:341–348. doi:10.1378/chest.129.2.341. 16478850

[8] Lai CC, Tan CK, Chou CH, Hsu HL, Liao CH, Huang YT, Yang PC, Luh KT, Hsueh PR. 2010. Increasing incidence of nontuberculous mycobacteria, Taiwan, 2000–2008. Emerg Infect Dis 16:294–296. doi:10.3201/eid1602.090675. 20113563 PMC2958002

[9] Wang X, Li H, Jiang G, Zhao L, Ma Y, Javid B, Huang H. 2014. Prevalence and drug resistance of nontuberculous mycobacteria, Northern China, 2008–2011. Emerg Infect Dis 20:1252–1253. doi:10.3201/eid2007.131801. 24959839 PMC4073877

[10] Cheng A, Sun H-Y, Tsai Y-T, Lu P-L, Lee SS-J, Lee Y-T, Wang Y-C, Liu P-Y, Chien J-Y, Hsueh P-R, Chang S-Y, Wu U-I, Sheng W-H, Chen Y-C, Chang S-C. 2021. Longitudinal non-cystic fibrosis trends of pulmonary *Mycobacterium abscessus* disease from 2010 to 2017: Spread of the “globally successful clone” in Asia. ERJ Open Res 7:00191-2020. doi:10.1183/23120541.00191-2020. PMC7836708 33532483

[11] Tortoli E, Kohl TA, Brown-Elliott BA, Trovato A, Leão SC, Garcia MJ, Vasireddy S, Turenne CY, Griffith DE, Philley JV, Baldan R, Campana S, Cariani L, Colombo C, Taccetti G, Teri A, Niemann S, Wallace RJ, Cirillo DM. 2016. Emended description of *Mycobacterium abscessus, Mycobacterium abscessus* subsp. *abscessus* and *Mycobacterium abscessus* subsp. *bolletii* and designation of *Mycobacterium abscessus* subsp. *massiliense* comb. nov. Int J Syst Evol Microbiol 66:4471–4479. doi:10.1099/ijsem.0.001376. 27499141

[12] Johansen MD, Herrmann JL, Kremer L. 2020. Non-tuberculous mycobacteria and the rise of *Mycobacterium abscessus*. Nat Rev Microbiol 18:392–407. doi:10.1038/s41579-020-0331-1. 32086501

[13] Daley CL, Iaccarino JM, Lange C, Cambau E, Wallace RJ, Andrejak C, Böttger EC, Brozek J, Griffith DE, Guglielmetti L, Huitt GA, Knight SL, Leitman P, Marras TK, Olivier KN, Santin M, Stout JE, Tortoli E, van Ingen J, Wagner D, Winthrop KL. 2020. Treatment of nontuberculous mycobacterial pulmonary disease: an official ATS/ERS/ESCMID/IDSA clinical practice guideline: executive summary. Clin Infect Dis 71:e1–e36. doi:10.1093/cid/ciaa241. 32628747 PMC7768748

[14] Yoshida M, Sano S, Chien J-Y, Fukano H, Suzuki M, Asakura T, Morimoto K, Murase Y, Miyamoto S, Kurashima A, Hasegawa N, Hsueh P-R, Mitarai S, Ato M, Hoshino Y. 2021. A novel DNA chromatography method to discriminate *Mycobacterium abscessus* subspecies and macrolide susceptibility. EBioMedicine 64:103187. doi:10.1016/j.ebiom.2020.103187. 33446475 PMC7910664

[15] Bryant JM, Grogono DM, Greaves D, Foweraker J, Roddick I, Inns T, Reacher M, Haworth CS, Curran MD, Harris SR, Peacock SJ, Parkhill J, Floto RA. 2013. Whole-genome sequencing to identify transmission of *Mycobacterium abscessus* between patients with cystic fibrosis: a retrospective cohort study. Lancet 381:1551–1560. doi:10.1016/S0140-6736(13)60632-7. 23541540 PMC3664974

[16] Harris KA, Underwood A, Kenna DTD, Brooks A, Kavaliunaite E, Kapatai G, et al. 2015. Whole-genome sequencing and epidemiological analysis do not provide evidence for cross-transmission of *Mycobacterium abscessus* in a cohort of pediatric cystic fibrosis patients. Clin Infect Dis 60:1007–1016. 25452595 10.1093/cid/ciu967 PMC4357290

[17] Redondo N, Mok S, Montgomery L, Flanagan PR, McNamara E, Smyth EG, O'Sullivan N, Schaffer K, Rogers TR, Fitzgibbon MM. 2020. Genomic analysis of *Mycobacterium abscessus* complex isolates collected in Ireland between 2006 and 2017. J Clin Microbiol 58:e00295-20. doi:10.1128/JCM.00295-20. 32295892 PMC7315040

[18] Davidson RM, Hasan NA, Epperson LE, Benoit JB, Kammlade SM, Levin AR, Calado de Moura V, Hunkins J, Weakly N, Beagle S, Sagel SD, Martiniano SL, Salfinger M, Daley CL, Nick JA, Strong M. 2021. Population genomics of *Mycobacterium abscessus* from US cystic fibrosis care centers. Ann Am Thorac Soc 18:1960–1969. doi:10.1513/AnnalsATS.202009-1214OC. 33856965 PMC8641822

[19] Bryant JM, Grogono DM, Rodriguez-Rincon D, Everall I, Brown KP, Moreno P, Verma D, Hill E, Drijkoningen J, Gilligan P, Esther CR, Noone PG, Giddings O, Bell SC, Thomson R, Wainwright CE, Coulter C, Pandey S, Wood ME, Stockwell RE, Ramsay KA, Sherrard LJ, Kidd TJ, Jabbour N, Johnson GR, Knibbs LD, Morawska L, Sly PD, Jones A, Bilton D, Laurenson I, Ruddy M, Bourke S, Bowler IC, Chapman SJ, Clayton A, Cullen M, Daniels T, Dempsey O, Denton M, Desai M, Drew RJ, Edenborough F, Evans J, Folb J, Humphrey H, Isalska B, Jensen-Fangel S, Jönsson B, Jones AM, et al. 2016. Emergence and spread of a human-transmissible multidrug-resistant nontuberculous mycobacterium. Science 354:751–757. doi:10.1126/science.aaf8156. 27846606 PMC5142603

[20] Davidson RM, Hasan NA, Reynolds PR, Totten S, Garcia B, Levin A, Ramamoorthy P, Heifets L, Daley CL, Strong M. 2014. Genome sequencing of *Mycobacterium abscessus* isolates from patients in the United States and comparisons to globally diverse clinical strains. J Clin Microbiol 52:3573–3582. doi:10.1128/JCM.01144-14. 25056330 PMC4187745

[21] Ruis C, Bryant JM, Bell SC, Thomson R, Davidson RM, Hasan NA, van Ingen J, Strong M, Floto RA, Parkhill J. 2021. Dissemination of *Mycobacterium abscessus* via global transmission networks. Nat Microbiol 6:1279–1288. doi:10.1038/s41564-021-00963-3. 34545208 PMC8478660

[22] Tortoli E, Kohl TA, Trovato A, Baldan R, Campana S, Cariani L, Colombo C, Costa D, Cristadoro S, Di Serio MC, Manca A, Pizzamiglio G, Rancoita PMV, Rossolini GM, Taccetti G, Teri A, Niemann S, Cirillo DM. 2017. *Mycobacterium abscessus* in patients with cystic fibrosis: low impact of inter-human transmission in Italy. Eur Respir J 50:1602525. doi:10.1183/13993003.02525-2016. 28705942

[23] Yamashiro Y, Shimizu T, Oguchi S, Shioya T, Nagata S, Ohtsuka Y. 1997. The estimated incidence of cystic fibrosis in Japan. J Pediatr Gastroenterol Nutr 24:544–547. doi:10.1097/00005176-199705000-00010. 9161949

[24] Singh M, Rebordosa C, Bernholz J, Sharma N. 2015. Epidemiology and genetics of cystic fibrosis in Asia: in preparation for the next-generation treatments. Respirology 20:1172–1181. doi:10.1111/resp.12656. 26437683

[25] Bastian S, Veziris N, Roux A-L, Brossier F, Gaillard J-L, Jarlier V, Cambau E. 2011. Assessment of clarithromycin susceptibility in strains belonging to the *Mycobacterium abscessus* group by *erm*(41) and *rrl* sequencing. Antimicrob Agents Chemother 55:775–781. doi:10.1128/AAC.00861-10. 21135185 PMC3028756

[26] Kim H-Y, Kim BJ, Kook Y, Yun Y-J, Shin JH, Kim B-J, Kook Y-H. 2010. *Mycobacterium massiliense* is differentiated from *Mycobacterium abscessus* and *Mycobacterium bolletii* by erythromycin ribosome methyltransferase gene (erm) and clarithromycin susceptibility patterns. Microbiol Immunol 54:347–353. doi:10.1111/j.1348-0421.2010.00221.x. 20536733

[27] Nash KA, Brown-Elliott AB, Wallace RJ. 2009. A novel gene, *erm*(41), confers inducible macrolide resistance to clinical isolates of *Mycobacterium abscessus* but is absent from *Mycobacterium chelonae*. Antimicrob Agents Chemother 53:1367–1376. doi:10.1128/AAC.01275-08. 19171799 PMC2663066

[28] Brown-Elliott BA, Vasireddy S, Vasireddy R, Iakhiaeva E, Howard ST, Nash K, Parodi N, Strong A, Gee M, Smith T, Wallace RJ. 2015. Utility of sequencing the *erm*(41) gene in isolates of *Mycobacterium abscessus* subsp. *abscessus* with low and intermediate clarithromycin MICs. J Clin Microbiol 53:1211–1215. doi:10.1128/JCM.02950-14. 25653399 PMC4365201

[29] Wallace RJ, Meier A, Brown BA, Zhang Y, Sander P, Onyi GO, Böttger EC. 1996. Genetic basis for clarithromycin resistance among isolates of *Mycobacterium chelonae* and *Mycobacterium abscessus*. Antimicrob Agents Chemother 40:1676–1681. doi:10.1128/AAC.40.7.1676. 8807061 PMC163394

[30] Nessar R, Cambau E, Reyrat JM, Murray A, Gicquel B. 2012. *Mycobacterium abscessus*: a new antibiotic nightmare. J Antimicrob Chemother 67:810–818. doi:10.1093/jac/dkr578. 22290346

[31] Lopeman RC, Harrison J, Desai M, Cox JAG. 2019. *Mycobacterium abscessus*: environmental bacterium turned clinical nightmare. Microorganisms 7:90. doi:10.3390/microorganisms7030090. 30909391 PMC6463083

[32] Tettelin H, Davidson RM, Agrawal S, Aitken ML, Shallom S, Hasan NA, Strong M, de Moura VCN, De Groote MA, Duarte RS, Hine E, Parankush S, Su Q, Daugherty SC, Fraser CM, Brown-Elliott BA, Wallace RJ, Holland SM, Sampaio EP, Olivier KN, Jackson M, Zelazny AM. 2014. High-level relatedness among *Mycobacterium abscessus* subsp. *massiliense* strains from widely separated outbreaks. Emerg Infect Dis 20:364–371. doi:10.3201/eid2003.131106. 24565502 PMC3944860

[33] Doyle RM, Rubio M, Dixon G, Hartley J, Klein N, Coll P, Harris KA. 2020. Cross-transmission is not the source of new *Mycobacterium abscessus* infections in a multicenter cohort of cystic fibrosis patients. Clin Infect Dis 70:1855–1864. doi:10.1093/cid/ciz526. 31225586 PMC7156781

[34] Morimoto K, Nakagawa T, Asami T, Morino E, Fujiwara H, Hase I, Tsujimoto Y, Izumi K, Hayashi Y, Matsuda S, Murase Y, Yano R, Takasaki J, Betsuyaku T, Aono A, Goto H, Nishimura T, Sasaki Y, Hoshino Y, Kurashima A, Ato M, Ogawa K, Hasegawa N, Mitarai S. 2018. Clinico-microbiological analysis of 121 patients with pulmonary *Mycobacteroides abscessus* complex disease in Japan: an NTM-JRC study with RIT. Respir Med 145:14–20. doi:10.1016/j.rmed.2018.10.012. 30509703

[35] Yoshida S, Arikawa K, Tsuyuguchi K, Kurashima A, Harada T, Nagai H, Suzuki K, Iwamoto T, Hayashi S. 2015. Investigation of the population structure of *Mycobacterium abscessus* complex strains using 17-locus variable number tandem repeat typing and the further distinction of *Mycobacterium massiliense* hsp65 genotypes. J Med Microbiol 64:254–261. doi:10.1099/jmm.0.000016. 25596119

[36] Macheras E, Konjek J, Roux A-L, Thiberge J-M, Bastian S, Leão SC, Palaci M, Sivadon-Tardy V, Gutierrez C, Richter E, Rüsch-Gerdes S, Pfyffer GE, Bodmer T, Jarlier V, Cambau E, Brisse S, Caro V, Rastogi N, Gaillard J-L, Heym B. 2014. Multilocus sequence typing scheme for the *Mycobacterium abscessus* complex. Res Microbiol 165:82–90. doi:10.1016/j.resmic.2013.12.003. 24384536

[37] Li Q, Whalen CC, Albert JM, Larkin R, Zukowski L, Cave MD, Silver RF. 2002. Differences in rate and variability of intracellular growth of a panel of *Mycobacterium tuberculosis* clinical isolates within a human monocyte model. Infect Immun 70:6489–6493. doi:10.1128/IAI.70.11.6489-6493.2002. 12379735 PMC130434

[38] López B, Aguilar D, Orozco H, Burger M, Espitia C, Ritacco V, Barrera L, Kremer K, Hernandez-Pando R, Huygen K, van Soolingen D. 2003. A marked difference in pathogenesis and immune response induced by different *Mycobacterium tuberculosis* genotypes. Clin Exp Immunol 133:30–37. doi:10.1046/j.1365-2249.2003.02171.x. 12823275 PMC1808750

[39] Ribeiro SCM, Gomes LL, Amaral EP, Andrade MRM, Almeida FM, Rezende AL, Lanes VR, Carvalho ECQ, Suffys PN, Mokrousov I, Lasunskaia EB. 2014. *Mycobacterium tuberculosis* strains of the modern sublineage of the Beijing family are more likely to display increased virulence than strains of the ancient sublineage. J Clin Microbiol 52:2615–2624. doi:10.1128/JCM.00498-14. 24829250 PMC4097719

[40] Bronson RA, Gupta C, Manson AL, Nguyen JA, Bahadirli-Talbott A, Parrish NM, Earl AM, Cohen KA. 2021. Global phylogenomic analyses of *Mycobacterium abscessus* provide context for non cystic fibrosis infections and the evolution of antibiotic resistance. Nat Commun 12:5145. doi:10.1038/s41467-021-25484-9. 34446725 PMC8390669

[41] Bryant JM, Brown KP, Burbaud S, Everall I, Belardinelli JM, Rodriguez-Rincon D, Grogono DM, Peterson CM, Verma D, Evans IE, Ruis C, Weimann A, Arora D, Malhotra S, Bannerman B, Passemar C, Templeton K, MacGregor G, Jiwa K, Fisher AJ, Blundell TL, Ordway DJ, Jackson M, Parkhill J, Floto RA. 2021. Stepwise pathogenic evolution of *Mycobacterium abscessus*. Science 372:eabb8699. doi:10.1126/science.abb8699. 33926925 PMC7611193

[42] Cerritelli S, Springhorn SS, Lacks SA. 1989. DpnA, a methylase for single-strand DNA in the Dpn II restriction system, and its biological function. Proc Natl Acad Sci USA 86:9223–9227. doi:10.1073/pnas.86.23.9223. 2687877 PMC298466

[43] Cardoso AM, Martins de Sousa E, Viana-Niero C, Bonfim de Bortoli F, Pereira das Neves ZC, Leão SC, Junqueira-Kipnis AP, Kipnis A. 2008. Emergence of nosocomial *Mycobacterium massiliense* infection in Goiás, Brazil. Microbes Infect 10:1552–1557. doi:10.1016/j.micinf.2008.09.008. 18950729

[44] Kurtz S, Phillippy A, Delcher AL, Smoot M, Shumway M, Antonescu C, Salzberg SL. 2004. Versatile and open software for comparing large genomes. Genome Biol 5:R12. doi:10.1186/gb-2004-5-2-r12. 14759262 PMC395750

[45] Croucher NJ, Page AJ, Connor TR, Delaney AJ, Keane JA, Bentley SD, Parkhill J, Harris SR. 2015. Rapid phylogenetic analysis of large samples of recombinant bacterial whole genome sequences using Gubbins. Nucleic Acids Res 43:e15. doi:10.1093/nar/gku1196. 25414349 PMC4330336

[46] Stamatakis A. 2006. RAxML-VI-HPC: maximum likelihood-based phylogenetic analyses with thousands of taxa and mixed models. Bioinformatics 22:2688–2690. doi:10.1093/bioinformatics/btl446. 16928733

[47] Tonkin-Hill G, Lees JA, Bentley SD, Frost SDW, Corander J. 2019. Fast hierarchical Bayesian analysis of population structure. Nucleic Acids Res 47:5539–5549. doi:10.1093/nar/gkz361. 31076776 PMC6582336

[48] Tanizawa Y, Fujisawa T, Nakamura Y. 2018. DFAST: a flexible prokaryotic genome annotation pipeline for faster genome publication. Bioinformatics 34:1037–1039. doi:10.1093/bioinformatics/btx713. 29106469 PMC5860143

[49] Guy L, Kultima JR, Andersson SGE. 2010. genoPlotR: comparative gene and genome visualization in R. Bioinformatics 26:2334–2335. doi:10.1093/bioinformatics/btq413. 20624783 PMC2935412

