# Supplementary material for: Molecular Epidemiological Characteristics of Mycobacterium abscessus Complex Derived from Non-Cystic Fibrosis Patients in Japan and Taiwan
Source: Microbiol Spectr. 2022 Apr 21;10(3):e00571-22. doi: 10.1128/spectrum.00571-22 (PMC9248903; doi:10.1128/spectrum.00571-22)
Supplement: SUPPLEMENTAL FILE 1 — Supplemental material. Download spectrum.00571-22-s001.pdf, PDF file, 1.4 MB [file spectrum.00571-22-s001.pdf]

Supplemental material

Molecular epidemiological characteristics of *Mycobacterium abscessus* complex derived from non-cystic fibrosis patients in Japan and Taiwan

Mitsunori Yoshida, Jung-Yien Chien, Kozo Morimoto, Takeshi Kinjo, Akio Aono, Yoshiro Murase, Keiji Fujiwara, Yuta Morishige, Hiroaki Nagano, Ruwen Jou, Naoki Hasegawa, Manabu Ato, Yoshihiko Hoshino, Po-Ren Hsueh, Satoshi Mitarai

Supplemental Methods

DNA sequencing

All clinical isolates (n=220) were sub-cultured on 2% Ogawa egg slants or 7H10 agar plates supplemented with 10% OADC (BD BBL, MD, USA). The genomic DNA of each isolate was extracted and purified using a standard enzyme/organic solvent method (1). A WGS library was constructed using the QIAseq FX DNA Library Kit (QIAGEN, Hilden, Germany). Sequencing was performed on the standard NextSeq 550 platform (Illumina Inc., CA, USA) using the NextSeq Mid Output Kit 2.5 (300 cycles, Illumina Inc.) with 150-mer paired-end short reads according to the manufacturer's instructions. All raw read data of newly sequenced strains in this study (220 isolates) were deposited into the DNA Data Bank of Japan (DDBJ) and the National Centre for Biotechnology Information (NCBI) under BioProject accession number PRJDB10566.

Phylogenetic analyses based on core-genome variable positions of MABC isolates

The raw read data of each isolate were *de novo* assembled in the Shovill pipeline (https://github.com/tseemann/shovill) with default settings. The number of contigs, N50 values, and raw coverage of each isolate are listed in Table S4. The data were combined with the publicly available complete genome sequence of *M. abscessus* subsp. *abscessus* (ABS) ATCC19977 (2), *M. abscessus* subsp. *massiliense* (MAS) JCM15300 (3), and *M. abscessus* subsp. *bolletii* (BOL) BD (4). For phylogenetic analyses, we performed pairwise genome alignment between ATCC19977 and one of the clinical isolates using the MUMmer package (5) to identify conserved genomic regions among these isolates and the SNP sites located within these regions. We then combined all alignments into a whole-genome alignment, in which each position corresponded to that of the complete genome of ATCC19977 using custom Perl scripts. We identified 3,638,122 bp of the ATCC19977 genome that was conserved among all clinical isolates (core-genome) and

identified 235,540 variable positions within the core-genome. This alignment was used to infer recombination sites using Gubbins (6), and 8,358 recombinogenic sites were detected and removed from the alignment. The resulting alignment containing 240,429 recombination-free variable positions located in the core-genome region were used to reconstruct a maximum-likelihood tree using RAxML ver. 8.2.12 (7) with the General Time Reversible (GTR)-GAMMA model of nucleotide substitution and 300 bootstrap replicates. To confirm phylogeny-based subspecies identification, ANI values among all MABC isolates were calculated using fastANI with default settings (8). Multiple whole-genome alignments were used to investigate the genotypes of the *erm*(41) gene (MAB_2297, 2,345,955 bp to 2,346,476 bp in ATCC19977) and the *rrl* gene (MAB_r5052, 1,464,208 bp to 1,467,319 bp in ATCC19977) of all isolates using SeqKit (9).

After subspecies identification, the maximum-likelihood phylogeny within each ABS and MAS was constructed as described above. The complete genome sequences of ATCC19977 and JCM15300 were used as a reference, respectively. Alignments containing 76,114 and 48,718 recombination-free variable positions located on their core-genome regions (3,963,788 bp for ABS and 4,033,769 bp for MAS) were used to estimate maximum-likelihood phylogenies, respectively. The resulting phylogenetic trees were used to identify clusters (ABS-EA and MAS-EA clusters) in Japan and Taiwan using the TreeGubbins software with options '-s (significance cut-off level) 0.01, -p (number of permutations to run to test significance) 10000' (https://github.com/simonrharris/tree_gubbins). Only detected clusters from more than one location and greater than three isolates were considered ABS-EA/MAS-EA clusters to exclude possible point source outbreaks.

To assess the relationships among Japanese (around Tokyo and Okinawa), Taiwanese, and circulating clones of ABS or MAS in a total of eight countries, we combined our data set with

publicly available WGS data from 476 MABC clinical isolates (349 ABS and 127 MAS, Table S1). These were analyzed in three previous WGS-based epidemiological studies (10–12), with one strain per patient and known cluster information to which each isolate belongs (A. Floto, J. Parkhill, and J. Bryant, personal communication). All additional raw read data were obtained from the Sequence Read Archive (SRA) and assembled *de novo* using the Shovill pipeline. To measure the assembly quality of these additional isolates, we calculated the genome fraction (%) of each isolate, which is the total number of aligned bases in the reference genomes (ATCC19977 or JCM15300) using QUAST software (13). We used only isolates with genome fractions greater than 85% for downstream analyses. Alignments containing 102,613 and 58,975 recombination-free variable positions located on their core-genome regions (3,584,621 bp for ABS and 3,789,583 bp for MAS) were used to estimate maximum-likelihood phylogenies, and cluster analysis was performed as described above.

Analysis of lineage-associated genes

Lineage-associated genes were identified as described previously (14). In brief, we annotated all draft genomes and complete genome sequences of the three type strains (ATCC19977, JCM15300, and BD) of MABC using DFAST_core ver. 1.0.3 with default settings (15) and Roary software with default settings (16) to compute core genes or accessory genes. We identified accessory genes that were significantly associated with lineage using the Scoary (17).

Statistics

Statistical analyses were performed with R software (www.r-project.org). The R function multinom() was used to statistically assess the differences in the composition of MABC subspecies at the three locations. The R function wilcox.exact() was used to assess the statistical significance

of the differences in nucleotide diversities and pairwise SNP distances among MABC clinical isolates. Enrichments of macrolide resistance-associated mutations in ABS/MAS-EA clusters were statistically assessed using the R function fisher.test().

Supplemental Figures

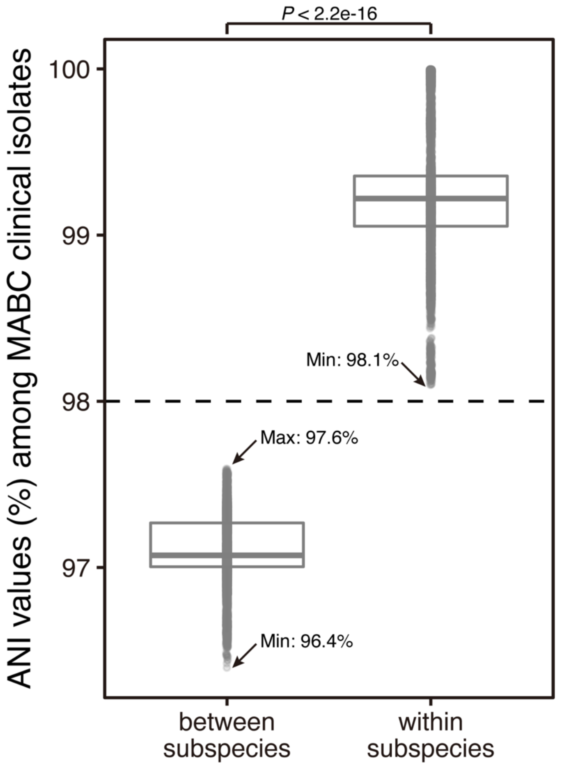

Figure S1. Average nucleotide identity (ANI) values among MABC clinical isolates from Japan and Taiwan. ANI values among 220 clinical isolates and three reference strains (ATCC19977, JCM15300, and BD) were measured for all strain pairs using fastANI (8). A boxplot indicates the distribution of ANI values within or between MABC subspecies. The Mann–Whitney U-test was performed to assess statistical significance between ANI values within or between the subspecies. The minimum ANI among MABC clinical isolates was 96.4%, all ANI values within the three subspecies were > 98% (minimum: 98.1%), while all ANI values between subspecies were < 98% (maximum: 97.6%). This indicates that the species and subspecies boundaries of the MABC clinical isolates were approximately 96% and 98% ANI, respectively, which is consistent with previous results (14).

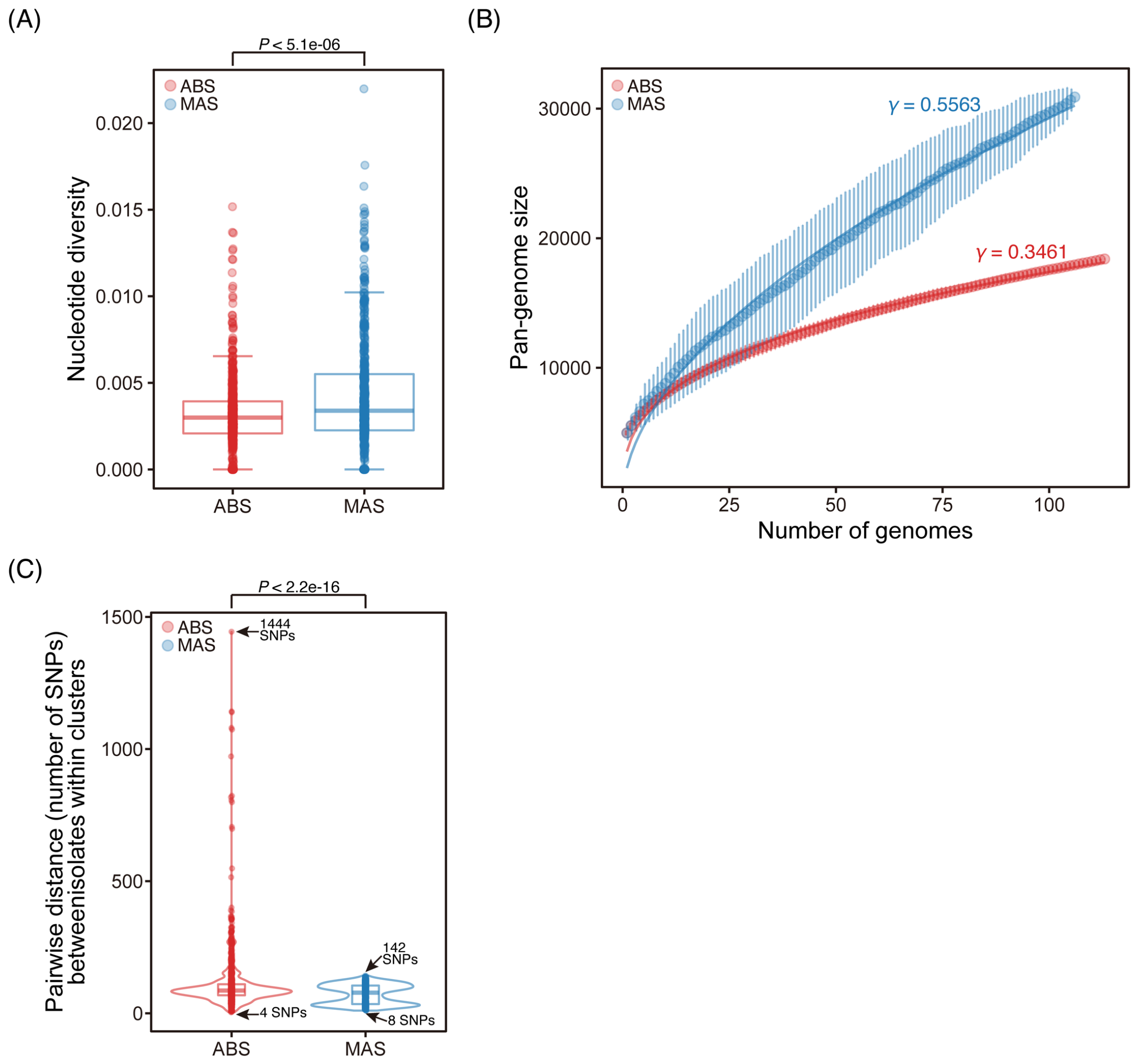

Figure S2. Populational comparison between ABS and MAS in Japan and Taiwan. (A) Nucleotide diversity of ABS or MAS clinical isolates in Japan and Taiwan was calculated for 10 kb nonoverlapping sliding windows of the genome alignments using the R package PopGenome (18). The Mann–Whitney U-test was used to assess the statistical significance of the differences between nucleotide diversities. (B) Comparison of pan-genome size between ABS and MAS clinical isolates in Japan and Taiwan. Orthologous gene families were identified using Roary software (16). γ denotes the parameter estimate obtained by data fitting to a power model $n = \kappa \times N^\wedge\gamma$, where n is the number of gene families, N is the number of sampled genomes, and κ and γ are coefficients. (C) Pairwise SNP distances among clinical isolates belonging to each of six ABS-EA or five MAS-EA clusters. Whole-genome alignments containing recombination-free variable positions located in core genomes were used to calculate the SNP distances using snp-dist (https://github.com/tseemann/snp-dists). The Mann–Whitney U-test was used to assess the statistical significance of the differences in pairwise SNP distances between clinical isolates comprising the ABS- and MAS-EA clusters.

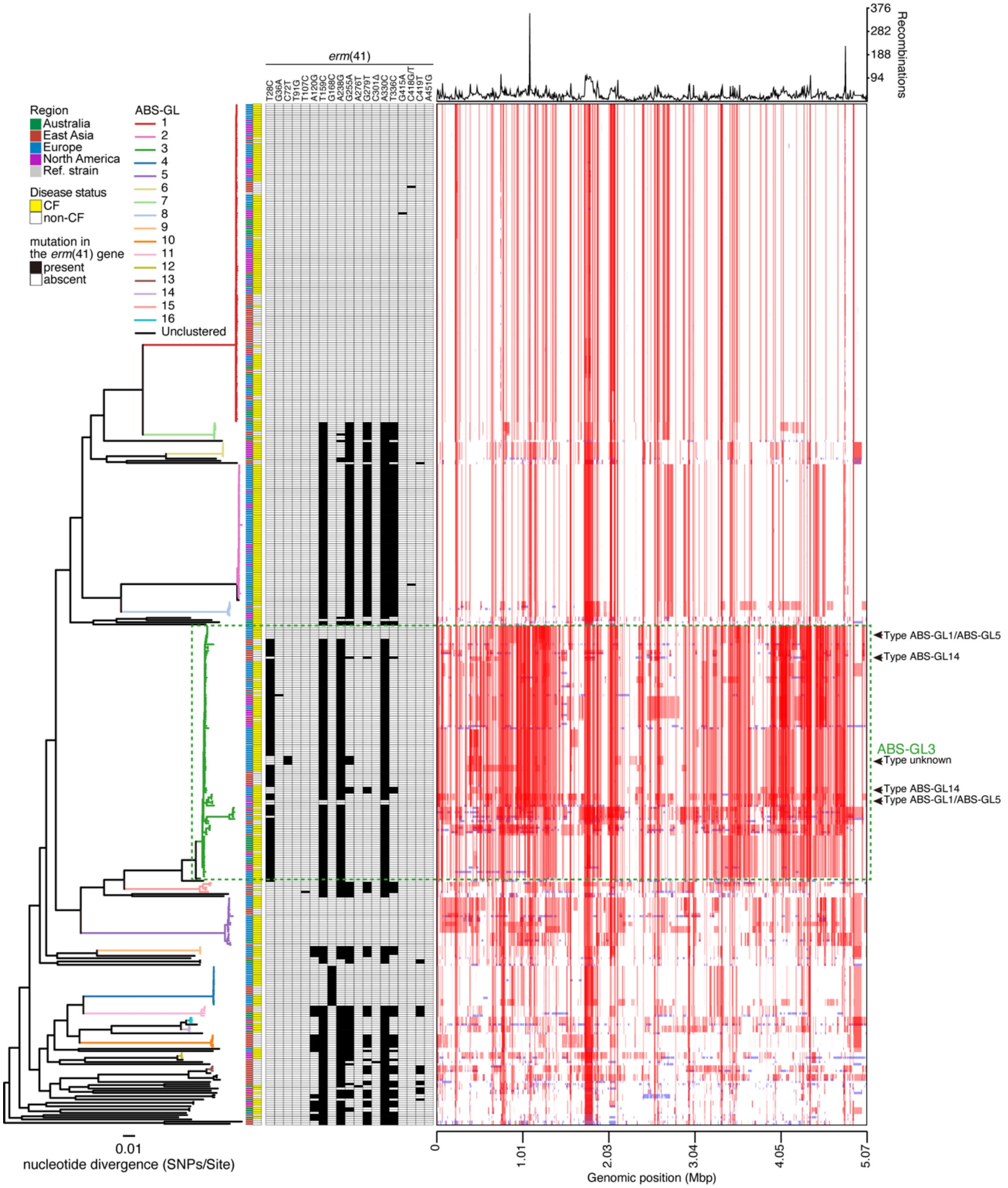

Figure S3. *erm*(41) gene among ABS clinical isolates. Overview of recombination events in a total of 461 ABS clinical isolates. Recombination events in internal branches (red boxes) were present in multiple clinical isolates and were shared through clonal descent, while those in the terminal branches (blue boxes) were clinical isolate-specific and represent independent recent acquisitions. Phylogenetic trees were estimated as described in Fig. 4. The presence and absence of mutations in the *erm*(41) gene, disease status (CF or non-CF) of corresponding patients, and the region where the clinical isolate was obtained are shown as Fig. 4.

Supplemental References

1. Sambrook J, Fritsch EF, Maniatis T. Molecular cloning: a laboratory manual. 2nd ed. In: Nolan C, editor. NY: Cold Spring Harbor Laboratory Press; 1989.
2. Ripoll F, Pasek S, Schenowitz C, Dossat C, Barbe V, Rottman M, et al. Non mycobacterial virulence genes in the genome of the emerging pathogen *Mycobacterium abscessus*. *PLoS One* 2009;4:e5660.
3. Sekizuka T, Kai M, Nakanaga K, Nakata N, Kazumi Y, Maeda S, et al. Complete Genome Sequence and Comparative Genomic Analysis of Mycobacterium massiliense JCM 15300 in the *Mycobacterium abscessus* Group Reveal a Conserved Genomic Island MmGI-1 Related to Putative Lipid Metabolism. In: Herrmann JL, editor. *PLoS One* 2014;9:e114848.
4. Yoshida M, Fukano H, Miyamoto Y, Shibayama K, Suzuki M, Hoshino Y. Complete genome sequence of a type strain of Mycobacterium abscessus subsp. bolletii, a member of the *Mycobacterium abscessus* complex. *Genome Announc* 2018;6:e01530-17.
5. Kurtz S, Phillippy A, Delcher AL, Smoot M, Shumway M, Antonescu C, et al. Versatile and open software for comparing large genomes. *Genome Biol* 2004;5:R12.
6. Croucher NJ, Page AJ, Connor TR, Delaney AJ, Keane JA, Bentley SD, et al. Rapid phylogenetic analysis of large samples of recombinant bacterial whole genome sequences using Gubbins. *Nucleic Acids Res* 2015;43:e15.
7. Stamatakis A. RAxML-VI-HPC: maximum likelihood-based phylogenetic analyses with thousands of taxa and mixed models. *Bioinformatics* 2006;22:2688-2690.

8. Jain C, Rodriguez-R LM, Phillippy AM, Konstantinidis KT, Aluru S. High throughput ANI analysis of 90K prokaryotic genomes reveals clear species boundaries. *Nat Commun* 2018;9:5114.

9. Shen W, Le S, Li Y, Hu F. SeqKit: A Cross-Platform and Ultrafast Toolkit for FASTA/Q File Manipulation. In: Zou Q, editor. *PLoS One* 2016;11:e0163962.

10. Bryant JM, Grogono DM, Rodriguez-Rincon D, Everall I, Brown KP, Moreno P, et al. Emergence and spread of a human-transmissible multidrug-resistant nontuberculous mycobacterium. *Science* 2016;354:751-757.

11. Bryant JM, Grogono DM, Greaves D, Foweraker J, Roddick I, Inns T, et al. Whole-genome sequencing to identify transmission of *Mycobacterium abscessus* between patients with cystic fibrosis: A retrospective cohort study. *Lancet* 2013;381:1551-1560.

12. Tettelin H, Davidson RM, Agrawal S, Aitken ML, Shallom S, Hasan NA, et al. High-level relatedness among *Mycobacterium abscessus* subsp. massiliense strains from widely separated outbreaks. *Emerg Infect Dis* 2014;20:364-371.

13. Gurevich A, Saveliev V, Vyahhi N, Tesler G. QUAST: Quality assessment tool for genome assemblies. *Bioinformatics* 2013;29:1072-1075.

14. Yoshida M, Sano S, Chien J-Y, Fukano H, Suzuki M, Asakura T, et al. A novel DNA chromatography method to discriminate *Mycobacterium abscessus* subspecies and macrolide susceptibility. *EBioMedicine* 2021;103187.doi:10.1016/j.ebiom.2020.103187.

15. Tanizawa Y, Fujisawa T, Nakamura Y. DFAST: a flexible prokaryotic genome annotation pipeline for faster genome publication. In: Hancock J, editor. *Bioinformatics* 2018;34:1037-1039.

16. Page AJ, Cummins CA, Hunt M, Wong VK, Reuter S, Holden MTG, et al. Roary: rapid large-scale prokaryote pan genome analysis. *Bioinformatics* 2015;31:3691-3693.

17. Brynildsrud O, Bohlin J, Scheffer L, Eldholm V. Rapid scoring of genes in microbial pan-genome-wide association studies with Scoary. *Genome Biol* 2016;17:238.

18. Pfeifer B, Wittelsbürger U, Ramos-Onsins SE, Lercher MJ. PopGenome: An efficient swiss army knife for population genomic analyses in R. *Mol Biol Evol* 2014;31:1929-1936.
